# [Retracted] Novel triazole analogs of apigenin-7-methyl ether exhibit potent antitumor activity against ovarian carcinoma cells via the induction of mitochondrial-mediated apoptosis

**DOI:** 10.3892/etm.2026.13192

**Published:** 2026-05-26

**Authors:** Yuyan Qi, Zhaoxia Ding, Yushuang Yao, Dehua Ma, Feifei Ren, Hongjuan Yang, Aiping Chen

Exp Ther Med 17:1670–1676, 2019; DOI: 10.3892/etm.2018.7138

Following the publication of this paper, it was drawn to the Editors’ attention by a concerned reader that the apoptotic assay data shown in Fig. 4 on p. 1674 were strikingly similar to data that had already appeared several years previously in an article written by different authors at different research institutes in the journal *Molecular Cancer*. Upon performing an independent analysis of the other data in this paper, it came to light that certain of the colony formation assay data in Fig. 3 and the flow cytometric plots in Fig. 5 were similarly featured in other articles written by different authors at different research institutes, some of which were published earlier than the above paper.

In view of the fact that the abovementioned data had already apparently been published previously, the Editor of *Experimental and Therapeutic Medicine* has decided that this paper should be retracted from the Journal. The authors were asked for an explanation to account for these concerns, but the Editorial Office did not receive a reply. The Editor apologizes to the readership for any inconvenience caused.

